# An epigenome-wide study of obesity in African American youth and young adults: novel findings, replication in neutrophils, and relationship with gene expression

**DOI:** 10.1186/s13148-017-0435-2

**Published:** 2018-01-05

**Authors:** Xiaoling Wang, Yue Pan, Haidong Zhu, Guang Hao, Yisong Huang, Vernon Barnes, Huidong Shi, Harold Snieder, James Pankow, Kari North, Megan Grove, Weihua Guan, Ellen Demerath, Yanbin Dong, Shaoyong Su

**Affiliations:** 1 Department of Pediatrics, Georgia Prevention Institute, Medical College of Georgia, Augusta University, HS-1640, Augusta, GA 30912 USA; 2 Cancer Center, Augusta University, Augusta, GA USA; 3 Department of Epidemiology, University of Groningen, University Medical Center Groningen, Groningen, the Netherlands; 4 Division of Epidemiology and Community Health, School of Public Health, University of Minnesota, Minneapolis, MN 55454 USA; 5 Department of Epidemiology, University of North Carolina at Chapel Hill, Chapel Hill, NC 27514 USA; 6 Human Genetics Center, School of Public Health, University of Texas Health Sciences Center at Houston, Houston, TX 77030 USA

**Keywords:** DNA methylation, Youth, African Americans, Obesity, Leukocytes, Neutrophils

## Abstract

**Background:**

We conducted an epigenome-wide association study (EWAS) on obesity in healthy youth and young adults and further examined to what extent identified signals influenced gene expression and were independent of cell type composition and obesity-related cardio-metabolic risk factors. Genome-wide DNA methylation data from leukocytes were obtained from 700 African Americans aged 14–36. We also measured genome-wide DNA methylation data from neutrophils as well as genome-wide gene expression data from leukocytes in a subset of samples (*n* = 188).

**Results:**

The EWAS identified 76 obesity-related CpG sites in leukocytes with *p* < 1 × 10^−7^. In silico replication in the ARIC study of 2097 African Americans aged 47–70 validated 54 CpG sites. Out of the 54 CpG sites, 29 associations with obesity were novel and 37 were replicated in neutrophils. Fifty one CpG sites were associated with at least one cardio-metabolic risk factor; however, the number reduced to 9 after adjustment for obesity. Sixteen CpG sites were associated with expression of 17 genes in *cis*, of which 5 genes displayed differential expression between obese cases and lean controls. We also replicated 71.5% of obesity-related CpG sites previously reported.

**Conclusion:**

In this study of youth and young adults, we identified 29 novel CpG sites associated with obesity and replicated the majority of the CpG sites previously identified. We further demonstrated that the majority of the obesity-related CpG sites in leukocytes were not driven by cell composition or obesity-related cardio-metabolic risk factors. We also provided the direct link between DNA methylation-gene expression-obesity for 5 genes.

**Electronic supplementary material:**

The online version of this article (10.1186/s13148-017-0435-2) contains supplementary material, which is available to authorized users.

## Background

Obesity is a complex disease resulting from interactions between genes and environmental factors that can be modified and/or mediated by epigenetic changes. DNA methylation is a pivotal and stable epigenetic mechanism, and DNA methylation levels at particular loci have been associated with obesity and its related cardio-metabolic traits [1]. Recently, four large-scale epigenome-wide association studies (EWAS) [2–5] in middle-aged and older adults identified multiple DNA methylation loci in blood leukocytes that were associated with body mass index (BMI). However, the high prevalence of obesity comorbidities and use of medications in middle-aged or older populations may hide or bias obesity-related DNA methylation changes. For this reason, we investigated 700 healthy African American youth and young adults. With genome-wide DNA methylation data from leukocytes in the full data set and genome-wide DNA methylation data from purified neutrophils and genome-wide gene expression data from leukocytes in a subset of 188 subjects, the present study has three objectives. First, we conducted an EWAS of obesity in youth and young adults to identify new signals and further validate the findings with replication in an independent cohort of 2097 middle-aged African Americans from the Atherosclerosis Risk in Communities (ARIC) study [2]. Second, for these obesity-related DNA methylation loci, we checked whether the associations can be replicated in one purified cell type (neutrophils), whether the associations were driven by obesity-related cardio-metabolic traits, and whether the DNA methylation status of these loci was associated with gene expressions. Third, we examined to what extent we can replicate the findings previously reported in middle-aged and older adults. The identification of obesity-related DNA methylation changes in youth and young adult may provide new insights into the mechanisms linking obesity to associated clinical conditions at the early stages of the disease process and may provide new targets for early prevention.

## Methods

### Subjects

A total of 700 African American youths and young adults aged 14–36 were included in the current study. These subjects were participants from 3 existing cohorts (for details, see Additional file 1): the Epigenetic Basis of Obesity-Induced Cardiovascular Disease and Type 2 Diabetes (EpiGO) study [6] (96 obese [BMI percentile ≥ 95%] vs. 92 lean controls [BMI percentile ≤ 50%] aged 14–20), the LACHY study [7] (284 participants from the general population aged 14–18), and the BP stress study [8] (228 participants from the general population aged 18–36). All of these participants were free of chronic or acute disease and not on daily prescription medication for treatment of diseases. All participants were recruited from the southeastern USA. Height and weight were measured by standard methods using a wall-mounted stadiometer and a scale, respectively. BMI was calculated as weight/height^2^, and BMI percentile was calculated according to their age, sex, height, and weight. These studies were approved by the Institutional Review Board of Augusta University and performed following the guidelines of the Declaration of Helsinki. Written informed consent was provided by all participants or by their parents if they were less than 18 years.

### DNA extraction and genome-wide DNA methylation

For the participants from the EpiGO study, DNA was extracted from both peripheral leukocytes and neutrophils using the QIAamp DNA Mini Kit (QIAGEN). Peripheral neutrophils were obtained using the approach previously described [9]. For the other participants, DNA was extracted from stored buffy coats using the same kit.

Genome-wide DNA methylation levels of all these samples were analyzed by the Illumina Infinium Human Methylation 450K Beadchip (Illumina Inc.). The Minfi package [10] and CPACOR (incorporating Control Probe Adjustment and reduction of global CORrelation) package [11] were used for initial quantification, data preprocessing, and quality control (QC). The key QC steps included the following: (1) Detectable probes were defined as the probes with detection *p* value < 1 × 10^−16^ in more than 95% samples; (2) Detectable samples were defined as the samples with more than 95% CpG sites having a detection *p* value < 1 × 10^−16^ and correct classification of gender based on the genome-wide DNA methylation data; (3) Probes on the X and Y chromosomes and the 65 SNP markers were excluded; (4) Illumina background correction and quantile normalization were applied to all intensity values, and beta value was further calculated and used as the index of CpG methylation levels; (5) A principal component analysis (PCA) of the control probe intensities (excluding negative control probes) was performed and the resulting PCs 1 to 30 were stored; (6) White blood cell sub-populations were estimated using the approach described by Houseman et al. [12]; and (7) A linear regression model was conducted for each CpG site with DNA methylation level as the dependent variable and the 30 PCs from the control probe intensities as well as the estimated cell compositions as the independent variables. The residuals were calculated and used as indices of DNA methylation levels in further analysis. The above steps were conducted for each cohort separately.

### RNA extraction and genome-wide gene expression assays

For the participants of the EpiGO study, RNA samples were extracted from the peripheral leukocytes stored in the RNA cell protection reagents (QIAGEN Inc.) using the QIAamp RNA mini Kit (QIAGEN Inc.). RNA concentration and purity were evaluated on a NanoDrop spectrophotometer 2000 (Thermo Scientific Inc.). RNA integrity (RIN) was evaluated on a Bioanalyzer 2100 (Agilent Inc.). The RIN scores of all these samples were greater than 8, indicating the high quality of RNA.

Genome-wide gene expression data were obtained using the Illumina HumanHT-12 v4 Expression BeadChip (Illumina Inc). This chip targets more than 48,000 probes that provide genome-wide coverage of well-characterized genes, gene candidates, and splice variants. The Genome-Studio Gene Expression Module (Illumina Inc.) was used for initial quantification, and the lumi package [13] was used for data preprocessing and QC. The key QC steps included the following: (1) Probes with detection *p* value < 0.05 in more than 50% of the samples were defined as “present”; (2) Log transformation and quartile normalization were applied to the gene expression data; and (3) A linear regression model was conducted for each probe with gene expression level as the dependent variable and batches (16 batches with 12 samples in each batch) as the independent variables. The residuals were calculated and used as the indices of gene expression levels in further analysis.

### Statistical analysis

The R package Limma [14] was used for the identification of differentially methylated CpG sites and differentially expressed genes related to the obesity phenotypes.

For DNA methylation analysis on peripheral leukocytes, the residual derived from the regression of each single CpG site methylation level (beta) on cell compositions and PCs from the control probes was used as dependent variable with age and sex as covariates. The test of interest was group (obese vs. lean) in the EpiGO study and BMI in the LACHY and BP stress cohort. Meta-analysis across the three cohorts was conducted using METAL [15] by converting the direction of effect and *p* value observed in each study into a signed *Z* score. This approach is very flexible and allows results to be combined when the *β*-coefficients and standard errors from individual studies are in different units. To account for multiple testing, a standard Bonferroni correction for the 473,788 CpG sites gives *p* < 1 × 10^−07^ as the significance threshold.

For DNA methylation analysis on peripheral neutrophils, the residual derived from the regression of each single CpG site methylation level on PCs from the control probes was used as dependent variable with age and sex as covariates. The test of interest was group (obese vs. lean).

For gene expression analysis on peripheral leukocytes, the residual derived from the regression of each probe gene expression level on batches was used as dependent variable with age and sex as covariates. The test of interest was group (obese vs. lean).

The differentially methylated CpG sites identified for obesity and BMI in peripheral leukocytes with a *p* value< 1 × 10^−07^ were carried forward for replication in the African American participants of the ARIC study [2] (*n* = 2097, 64% females, age range from 47 to 70). Leukocyte DNA and the same Illumina 450K platform for methylation analysis were used in ARIC study. Replication was defined as consistent direction of the β-coefficient and FDR < 0.05 in the BMI-related CpG sites from the ARIC study. The replicated CpG sites were taken forward in the following analyses: First, they were checked in obesity-related CpGs identified from neutrophils. Replication was defined as consistent direction of the β-coefficient and FDR< 0.05 in neutrophils. Second, their associations with seven cardio-metabolic traits including SBP, fasting glucose, fasting insulin, fasting triglycerides (TG), fasting total cholesterol (TC), fasting HDL-cholesterol (HDLC), and fasting LDL-cholesterol (LDLC) were tested in EpiGO and LACHY cohorts with and without the adjustment of obesity status/BMI. A *p* value < 0.05 was defined as significant. Third, *cis*-regulation of these CpG sites of gene expression (within ± 250 kb of the CpG sites) was explored. Partial correlation of gene expression and DNA methylation was conducted with age, gender, and group (obese vs. lean) as covariates. A Bonferroni corrected *p* value < 0.05 according to the number of genes tested for each CpG site (range from 0 to 28) was defined as significant correlation. Obesity-related differentially expressed genes were defined as genes with their expression levels showing significant difference between obese cases and lean controls at *p* value < 0.05.

Pathway enrichment analysis was conducted for the genome-wide DNA methylation obtained from leukocytes using gene set enrichment analysis (GSEA) [16]. GSEA was performed on an unfiltered, ranked list of genes (ranked by the *p* values without consideration of directions), and a running-sum statistic was used to determine the enrichment of an a priori defined gene sets (pathways) based on the gene ranks. Statistical significance of pathway enrichment scores were ascertained by permutation testing over size-matched random gene sets and multiple testing was controlled by the false discovery rate (FDR). A FDR of 5% was used. Kyoto Encyclopedia of Genes and Genomes (KEGG) gene sets were used as the reference gene sets. The CpG site showing the most significant *p* value within a gene was used to represent the DNA methylation level of the gene.

We also checked whether we could replicate previously identified obesity-related CpG sites (*n* = 277) in our current study of youth and young adult [2–5]. Replication was defined as a consistent direction of the β-coefficient and FDR < 0.05.

## Results

### Discovery meta-analysis in the youth and young adult

**Fig. 1:**
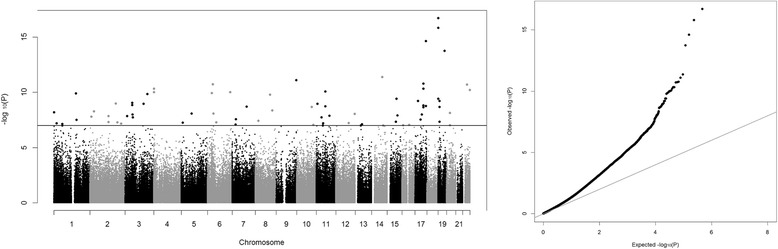
Left panel: Manhattan plot of the genome-wide DNA methylation analysis in youths and young adults. The dotted line indicates the Bonferroni threshold of 1E−07 for significance. Right panel: QQ plot of the genome-wide DNA methylation analysis in youth and young adults

Table 1 lists the general characteristics of the subjects. We identified 76 CpG sites significantly associated (*p* < 1 × 10^−07^) with obesity in the meta-analysis of the three cohorts (Manhattan and QQ-plot, Fig. 1; Additional file 2: Table S1). Obesity was positively associated with the methylation of 65 CpG sites and negatively associated with the methylation of 11 CpG sites. The top CpG site (cg12170787, *p* = 1.13 × 10^−16^) locates in the intron 5 of the *SBNO2* gene (Strawberry Notch Homolog 2). Two more CpG sites (cg18608055, *p* = 3.69 × 10^−16^; cg07573872, *p* = 1.12 × 10^−09^) locating in the same region also passed the threshold for genome-wide significance. CpG site cg07573872 is located in a CpG island shore region and has been reported to be associated with BMI previously [2]. CpG sites cg18608055 and cg12170787 are 4524 bp and 4623 bp away from cg07573872 (Additional file 3: Figure S1). The *SBNO2* gene is expressed in peripheral leukocytes and is a key player in the IL-10-regulated anti-inflammatory signaling pathway [17].

**Table 1:** General characteristics of the subjects

General characteristics	EpiGO		LACHY	BP stress cohort
	Lean	Obese		
*N*	92	96	284	228
Female (%)	50.0	51.0	50.0	57.5
Age (years)	17.7 ± 1.7	17.7 ± 1.8	16.2 ± 1.3	27.8 ± 3.3
Age range (years)	14.0–20.9	14.1–21.0	13.8–19.0	18.9–35.6
BMI (kg/m^2^)	18.8 ± 1.3	39.8 ± 6.8	24.1 ± 5.6	31.4 ± 8.6
BMI range (kg/m^2^)	15.0–21.7	28.1–70.1	16.4–45.9	17.6–59.6
BMI percentile (%)	18.7 ± 11.0	98.8 ± 1.1	65.7 ± 28.3	–
BMI percentile range (%)	0.8–41.7	95.0–99.9	2.3–99.7	–

### In silico replication with ARIC study

Of the 76 CpG sites significantly associated with obesity in our discovery meta-analysis, 54 replicated (FDR < 0.05) in the ARIC study (Table 2), of which 33 survived Bonferroni correction (*p* < 6.58 × 10^−4^). These 54 CpG sites annotated to 45 genes. In addition to *SBNO2* which exhibits 3 significant CpG sites, *SOCS3* (suppressor of cytokine signaling 3: cg18181703, cg04610187, and cg10508317), *CISH* (Cytoking-Inducible Src-Homology 2 –containing protein: cg21585138 and cg23005227), and *VMP1* (Vacuole Membrane Protein: cg16936953, cg12054453, cg24174557, cg18942579, and cg010409343) also have 2 or more significant CpG sites. The locations of these CpG sites within each gene and their correlations were provided in Additional file 3: Figure S1. Out of the 54 CpG sites associated with obesity, 29 CpG sites (annotated to 25 genes) were novel signals (highlighted in gray in Table 2).

**Table 2:**
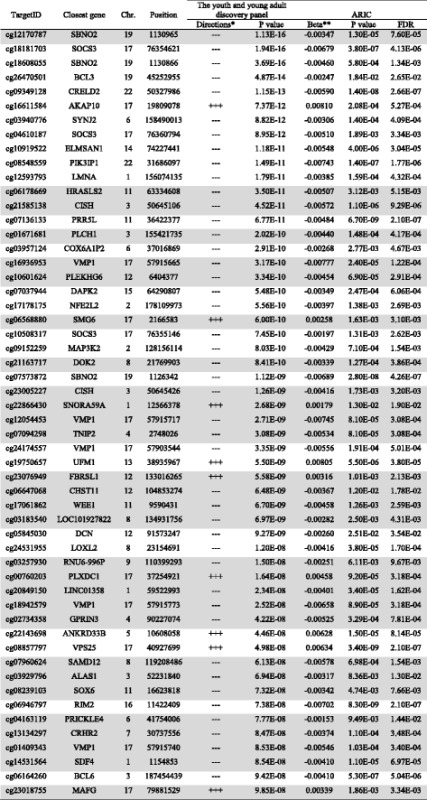
Validated CpG sites in the ARIC study

Newly discovered associations are highlighted in gray

*“+” indicates that DNA methylation levels increase with obesity status or BMI. The order is Epigo, LACHY, and BP stress cohort

**DNA methylation changes with 1 unit increase in BMI

### Replication of obesity-related CpG sites identified from mixed cells in purified neutrophils

The 54 CpG sites were taken forward to check whether the associations with obesity could be validated in one purified cell type: neutrophils. The results were listed in Additional file 2: Table S2. Thirty-seven CpG sites were found to be associated with obesity in neutrophils (FDR < 0.05 and consistent directions of effect), confirming that for the majority of the CpG sites (68.5%), the significant associations with obesity are not driven by potential cell compositions in leukocytes. For the 17 CpG sites that were not validated in neutrophils, it is difficult to determine whether these signals are driven by cell compositions or that neutrophils are not the biologically relevant cell type to target. The fact that 6 of the 17 CpG sites were hypermethylated (i.e., beta > 0.8) in neutrophils compared with 0 in the 37 replicated CpG sites (*p* = 1.2E-4) indicated that at least for some of the obesity-related CpG sites which could not be validated in neutrophils, neutrophils might not be the relevant cell type to target.

### Association with cardio-metabolic phenotypes

**Fig. 2:**
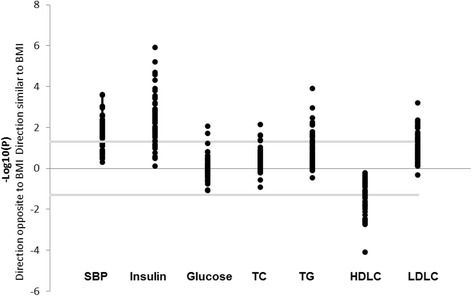
Manhattan plot depicting the − log_10_(*p* values) and effect direction (respectively to obesity) of the associations between the 55 replicated CpG sites and each cardiometabolic phenotype. The lines indicate the threshold of 0.05 for significance

We examined the associations between the 54 replicated CpG sites and 7 cardiometabolic traits (SBP, fasting insulin, fasting glucose, fasting lipid panel including TG, TC, HDLC, and LDLC). Without the adjustment of obesity or BMI, we observed 148 significant associations (*p* < 0.05, Additional file 2: Table S3) with 39 for SBP, 45 for insulin, 1 for glucose, 14 for TG, 5 for TC, 25 for HDLC, and 19 for LDLC. CpGs that were significantly associated with higher SBP, insulin, glucose, TG, TC, and LDLC were also associated with higher BMI levels. For HDLC, CpGs were associated with lower BMI levels (Fig. 2). After the adjustment of obesity or BMI, only 11 associations remained significant (Additional file 2: Table S4). The results indicate that the majority of the associations of these CpG sites with cardiometabolic phenotypes are driven by obesity.

### Gene expression analyses

**Fig. 3:**
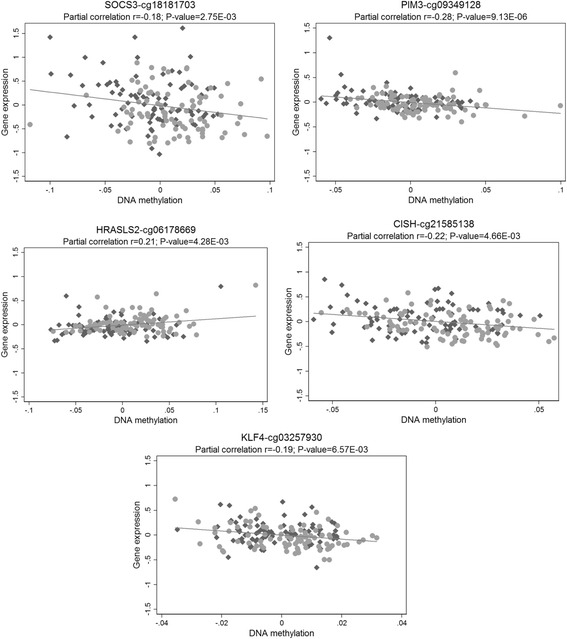
Partial correlations between DNA methylation and gene expression. Covariates included age, sex, and obesity status. Gray dots represent the lean group and black diamonds represent the obese group. For the SOCS3 gene, similar correlation was observed for cg04610187 (*r* = − 0.20, *p* = 1.77E−03); for the CISH gene, similar correlation was observed for cg23005227 (*r* = − 0.21, *p* = 7.14E−03)

Of the 54 CpG sites, 16 were significantly associated with expression of 17 unique genes in cis (Bonferroni corrected *p* value < 0.05) (Additional file 2: Table S5). Furthermore, of these 17 genes, the expression levels of 5 genes were significantly associated with obesity (*p* < 0.05) (Additional file 2: Table S5). Figure 3 shows the corresponding relationships between DNA methylation and gene expression for these 5 genes. With the exception of the correlation between cg06178669 and *HRASLS2* (HRAS-like suppressor 2), increased methylation was associated with decreased gene expressions. For *SOCS3*, *CISH*, *PIM3* (Pim-3 proto-oncogene, serine/threonine kinase), and *KLF4* (Kruppel-like factor 4), obesity was associated with decreased methylation and increased gene expression, while for *HRASLS2*, obesity was associated with decreased methylation and decreased gene expression (Additional file 2: Table S5).

### Pathway analyses

The pathway analyses yielded significant (FDR < 0.05) enrichment of 33 KEGG pathways for obesity-related DNA methylation changes in peripheral leukocytes (Additional file 2: Table S6). These pathways represent processes involved in inflammatory pathways (e.g., cell adhesion molecules, JAK-STAT signaling pathway, intestinal immune network for IGA production), autoimmune response (e.g., type 1 diabetes, allograft rejection, graft versus host disease), and obesity-related comorbidities (e.g. type 2 diabetes, vascular smooth muscle contraction, cancer, and asthma).

### Replication of previously reported CpG sites for obesity in middle-aged and elderly adults

We attempted to replicate the previously reported CpG sites for obesity. At the time this study was conducted, 4 large EWASs (with replication cohorts) for obesity were available: the ARIC study by Demerath E et al. [2] (reported 37 CpG sites), the Cardiogenics Consortium study by Dick K et al. [4] (reported 3 CpG sites), the GOLDN study by Aslibekyan S et al. [5] (reported 8 CpG sites), and the study reported by Wahl S et al. [3] (reported 254 CpG sites). These 4 studies resulted in 277 unique CpG sites associated with obesity. Out of these 277 CpG sites, 198 (71.5%) could be replicated with consistent direction of effect and a FDR < 0.05 in our study (Additional file 2: Table S7), indicating that majority of the obesity-related CpG sites identified in middle-aged and elderly adults already show changes in youth and young adulthood.

## Discussion

In this epigenome-wide association study of African American youth and young adults, we identified 29 novel CpG sites associated with obesity and the replicated majority of the CpG sites previously identified in middle-aged and older adults. We further demonstrated that majority of the obesity-related CpG sites identified from leukocytes are not driven by cell compositions or obesity-related cardio-metabolic risk factors. We also provided direct links between DNA methylation, gene expression, and obesity status for 7 CpG sites in 5 genes.

Unlike genetic markers that are the same in all cells and unlikely to change over a lifetime, epigenetic regulation is tissue specific and plastic (i.e., findings may reflect the consequence rather than the cause of the disease) [18]. In addition to availability, the choice of leukocytes in this study as well as in the previous epigenome-wide association studies of obesity in middle-aged and elder people is based on the fact that obesity is characterized by a state of chronic, low-grade inflammation [19]. With the established role of epigenetic regulation in shaping the immune and inflammatory response [20], identification of the epigenetic markers specifically involved in obesity-induced inflammation has the potential to provide novel insight into the pathogenesis of obesity-related disorders. The choice of leukocytes also assumes that the observed epigenetic changes will reflect consequences rather than causes of obesity. Although the cross-sectional design of the current and previous studies [2–5] does not provide information on the direction of causality, the Mendelian randomization model used in one [3] of the previous adult studies strongly suggested that obesity-related DNA methylation changes identified in peripheral leukocytes are predominately the consequence rather than the cause of obesity. One concern in analyzing epigenetic changes in peripheral leukocytes in obese individuals is that the effect of obesity itself on the immune system can be hidden or biased by the coexistence of obesity-related diseases and the use of medication, which is very common in middle-aged and elderly individuals. To avoid this problem, in this study, we focused on youth and young adults. This is a population having shown obesity-related metabolic risk that has not developed into clinical disease yet. We did observe that many obesity-related CpG sites were associated with cardiometabolic traits. However, after the adjustment of obesity, this number decreased dramatically, indicating that a large majority of the associations of these CpG sites with cardiometabolic phenotypes is driven by obesity.

In comparing with the previous studies in middle-aged adults and elderly [2–5], we identified 29 novel CpG sites (annotated to 25 genes). We were also able to present triangular relationships between DNA methylation, gene expression, and obesity at 5 genes including *SOCS3*, *CISH*, *PIM3*, *KLF4*, and *HRASLS2*. With the exception of *HARSLS2*, obesity was associated with decreased DNA methylation level and increased gene expressions. Interestingly, both *SOCS3* and *CISH* are involved in the JAK-STAT signaling pathway and are the key negative regulators of the activation of this pathway [21]. The JAK-STAT signaling pathway is activated during inflammation and used by a variety of cytokines. The JAK-STAT signaling pathway activates its own suppressors: suppressors of cytokine signaling (SOCS) molecules including SOCS1-7 and CISH. This negative feedback control is essential for the effective dissipation of cytokine signaling to prevent excessive inflammation and detrimental effect on other signaling pathways. However, the increased levels of SOCS proteins can induce insulin resistance in peripheral organs [22] and leptin resistance in the central nervous system [23]. There are very limited studies on *PIM3*, *KLF4*, and *HARSLS2*. However, PIM1, a protein from the same family and structurally and functionally similar to PIM3, is also primarily involved in the JAK/STAT signaling pathway. PIM-1 transcription is initiated by STAT factors and can also bind to regulators of the JAK/STAT pathway, resulting in a negative feedback loop [24]. Taken together, the activation of the JAK/STAT signaling pathway in obesity transcriptionally upregulates its own suppressors through DNA methylation mechanisms. On the one hand, the increased production of negative regulators can prevent excessive inflammation, while on the other hand, these increased negative feedback products may increase the risk of obesity-related disorders, a compensatory mechanism that has been observed in the development of many diseases.

Our study in healthy youth and young adults also replicated 71.5% of obesity-related CpG sites previously reported in middle-aged and elderly adults, indicating that the majority of the obesity-related CpG sites already show changes in adolescence. It will be interesting to find out the age of onset of these changes. In a recent study exploring this question on the first EWAS-identified gene (*HIF3A*), Pan et al. [25] observed that DNA methylation levels at the three previously described *HIF3A* CpG sites were already associated with greater weight and adiposity at birth. On the other hand, the strong consistency of obesity-related DNA methylation signals across ethnic groups (African Americans in this study comparing with Caucasians, Asians, and African Americans in previous studies) and age emphasizes the importance of conducting large-scale epigenome-wide meta-analysis similar to what has been done in the GWAS. This action will discover more obesity-related epigenome-wide changes and enable the recognition of the overall picture of the role of DNA methylation in obesity and its related disorders.

One possible concern in these EWAS studies on obesity using peripheral blood is the heterogeneity of leukocytes since different cell populations have distinct epigenetic signatures [26]. This concern was dramatically reduced with the development of algorithms estimating the cell subset compositions based on genome-wide DNA methylation data [12]. Our study further confirms this through successfully replication of 68.5% of the obesity-related CpG sites from leukocytes in one purified cell type: neutrophils. The reason that neutrophils were selected is that previous studies including ours [9, 27] have observed that obesity is consistently associated with neutrophilia, manifesting not only by elevated neutrophil count but also increased percentage. However, we believe that replication of the findings from leukocytes in a variety of other single cell types remains important to guide further functional in vitro studies.

Several limitations to the present study need to be recognized. First, although the study by Wahl S et al. [3] in adults have shown that obesity-related DNA methylation changes identified in peripheral leukocytes are predominately the consequence rather than the cause of obesity, we cannot test the direction of the causality of these newly identified obesity-related CpG sites in youth and young adults due to the cross-sectional design and the lack of genotype data. Second, although 71% of the signals from the discovery cohort were validated in the replication cohort, the lack of replication of other signals might be due to the age difference between the discovery cohort and the replication cohort. Third, due to the lack of genotype data, we cannot adjust the potential bias resulted from ancestry compositions.

## Conclusion

In summary, this EWAS study in healthy youth and young adult confirmed the majority of previously identified obesity-related DNA methylation loci in middle-aged and elderly adults and identified 29 novel CpG sites associated with obesity. The findings from the current study have the potential to enable development of new strategies for early prevention and treatment.

## Additional files

Additional file 1: Detailed description of the participants for each study. (DOCX 22 kb)

Additional file 2: Table S1. CpG sites with *p* < 1E−7 in this youth and young adult study. **Table S2.** Performance of the 55 CpG sites in neutrophils. **Table S3.** Association of the 55 CpG sites with cardiometabolic traits without adjustment of obesity/BMI. **Table S4.** The significant associations between CpG sites and metabolic traits after the adjustment of obesity/BMI. **Table S5.** DNA methylation, gene expression, and obesity. **Table S6.** Significantly enriched pathways (FDR < 0.05). **Table S7.** Replication of previous reported CpG sites identified in middle-aged and elderly population. (XLSX 64 kb)

Additional file 3: Figure S1. Positions and correlations of the multiple CpG sites in SBNO2, SOCS3, VMP1, and CISH genes. (PDF 425 kb)

## Abbreviations

ARIC: Atherosclerosis Risk in Communities
BMI: Body mass index
BP: Blood pressure
CISH: Cytoking-Inducible Src-Homology 2–containing protein
CPACOR: Incorporating Control Probe Adjustment and reduction of global CORrelation
CpG: 5'—C—phosphate—G—3'
DNA: Deoxyribonucleic acid
EpiGO: Epigenetic Basis of Obesity-Induced Cardiovascular Disease and Type 2 Diabetes
EWAS: Epigenome-wide association study
FDR: False discovery rate
GOLDN: Genetics of Lipid Lowering Drugs and Diet Network
GSEA: Gene set enrichment analysis
GWAS: Genome-wide association study
HDLC: Fasting HDL-cholesterol
HIF3A: Hypoxia-inducible factor 3 alpha subunit
HRASLS2: HRAS-like suppressor 2
IL-10: Interleukin-10
JAK-STAT: Janus kinase/signal transducers and activators of transcription
KEGG: Kyoto Encyclopedia of Genes and Genomes
KLF4: Kruppel-like factor 4
LACHY: Lifestyle, Adiposity, and Cardiovascular Health in Youth
LDLC: Fasting LDL-cholesterol
PCA: Principal component analysis
PIM1: Pim-1 proto-oncogene, serine/threonine kinase
PIM3: Pim-3 proto-oncogene, serine/threonine kinase
QC: Quality control
RIN: RNA integrity
SBNO2: Strawberry notch homolog 2
SBP: Systolic blood pressure
SNP: Single-nucleotide polymorphism
SOCS: Suppressors of cytokine signaling
SOCS3: Suppressor of cytokine signaling 3
TC: Fasting total cholesterol
TG: Fasting triglycerides
VMP1: Vacuole membrane protein
