# Supplementary material for: An epigenome-wide study of obesity in African American youth and young adults: novel findings, replication in neutrophils, and relationship with gene expression
Source: Clin Epigenetics. 2018 Jan 5;10:3. doi: 10.1186/s13148-017-0435-2 (PMC5756368; doi:10.1186/s13148-017-0435-2)
Supplement: Supplementary file 1 — Detailed description of the participants for each study. (DOCX 22 kb) [file 13148_2017_435_MOESM1_ESM.docx]

**Participants**

The 700 participants were from the following three studies:

*Epigenetic Basis of Obesity Induced Cardiovascular Disease and Type 2 Diabetes (EpiGO) study* [[1](#_ENREF_1)]. The EpiGO study was established in 2011 with the goal of identifying methylation changes involved in the pathogenesis of obesity and its related co-morbidities. The EpiGO study in total enrolled 351 obese (BMI percentile ≥95%) and 435 lean (BMI percentile ≤50%) youth aged 14 to 20 years with 70% of African-Americans (AA), 30% of European-Americans (EA), 47% of males and 53% of females. All the subjects were recruited from the Augusta, Georgia area from 2011-2015. The youths were free of any acute or chronic illness, and did not take daily prescription medication to treat diseases. A total of 192 African-American participants (96 obese vs. 96 lean, 50% of females) were selected for the current study. After quality control, genome wide DNA methylation data and gene expression data in leukocytes and genome wide DNA methylation data in neutrophils were available for 188 subjects (96 obese vs. 92 controls).

*Lifestyle, Adiposity, and Cardiovascular Health in Youth (LACHY) study* [[2](#_ENREF_2)]: The LACHY study was established in 2000 and finished in 2004 with the goal of determining the relations of fatness and fitness to cardiovascular disease (CVD) risk factors in the juvenile years. It consisted of 756 youths aged from 14 to 18 years recruited from general population, with roughly equal numbers of AA and EA of both genders. All participants were recruited from the Augusta, Georgia area. The youths were apparently healthy and did not take daily prescription medication to treat diseases. In this study, all AA participants with buffy coat available (n=286) were selected for the current study. After quality control, genome wide DNA methylation data in leukocytes were available for 284 subjects.

*Blood Pressure (BP) stress study* [[3](#_ENREF_3)]: The BP stress study is a longitudinal study, which was established in 1989 with the goal of exploring the development of cardiovascular risk factors. It consisted of 396 EA and 349 AA health youths aged from 7 to 16 years at baseline. All participants were recruited from the general population in the Augusta, Georgia area. The evaluation was conducted annually from 1989 to 2001 (visit 1-10), and every 1.5 years from 2002 to 2007 (visit 11-14). Most recently two more visits were conducted from 2008 to 2012 (visit 15-16). Blood samples were collected from visit 9. In the current study, all the AA participants in visit 15 with buffy coat available were selected for the current study (n=228). Genome wide DNA methylation data for all the subjects passed the quality control.

**Reference**

1. Xu X, Su S, Barnes VA, et al. A genome-wide methylation study on obesity: differential variability and differential methylation. Epigenetics : official journal of the DNA Methylation Society 2013;8:522-533

2. Gutin B, Johnson MH, Humphries MC, et al. Relationship of visceral adiposity to cardiovascular disease risk factors in black and white teens. Obesity (Silver Spring) 2007;15:1029-1035

3. Wang X, Poole JC, Treiber FA, et al. Ethnic and gender differences in ambulatory blood pressure trajectories: results from a 15-year longitudinal study in youth and young adults. Circulation 2006;114:2780-2787
