# Supplementary material for: An epigenome-wide study of obesity in African American youth and young adults: novel findings, replication in neutrophils, and relationship with gene expression
Source: Clin Epigenetics. 2018 Jan 5;10:3. doi: 10.1186/s13148-017-0435-2 (PMC5756368; doi:10.1186/s13148-017-0435-2)
Supplement: Supplementary file 3 — Positions and correlations of the multiple CpG sites in SBNO2, SOCS3, VMP1, and CISH genes. (PDF 425 kb) [file 13148_2017_435_MOESM3_ESM.pdf]

Additional file 3:

Figure S1. Positions and correlations of the multiple CpG sites in SBNO2, SOCS3, VMP1 and CISH genes

CpG sites in SBNO2 gene

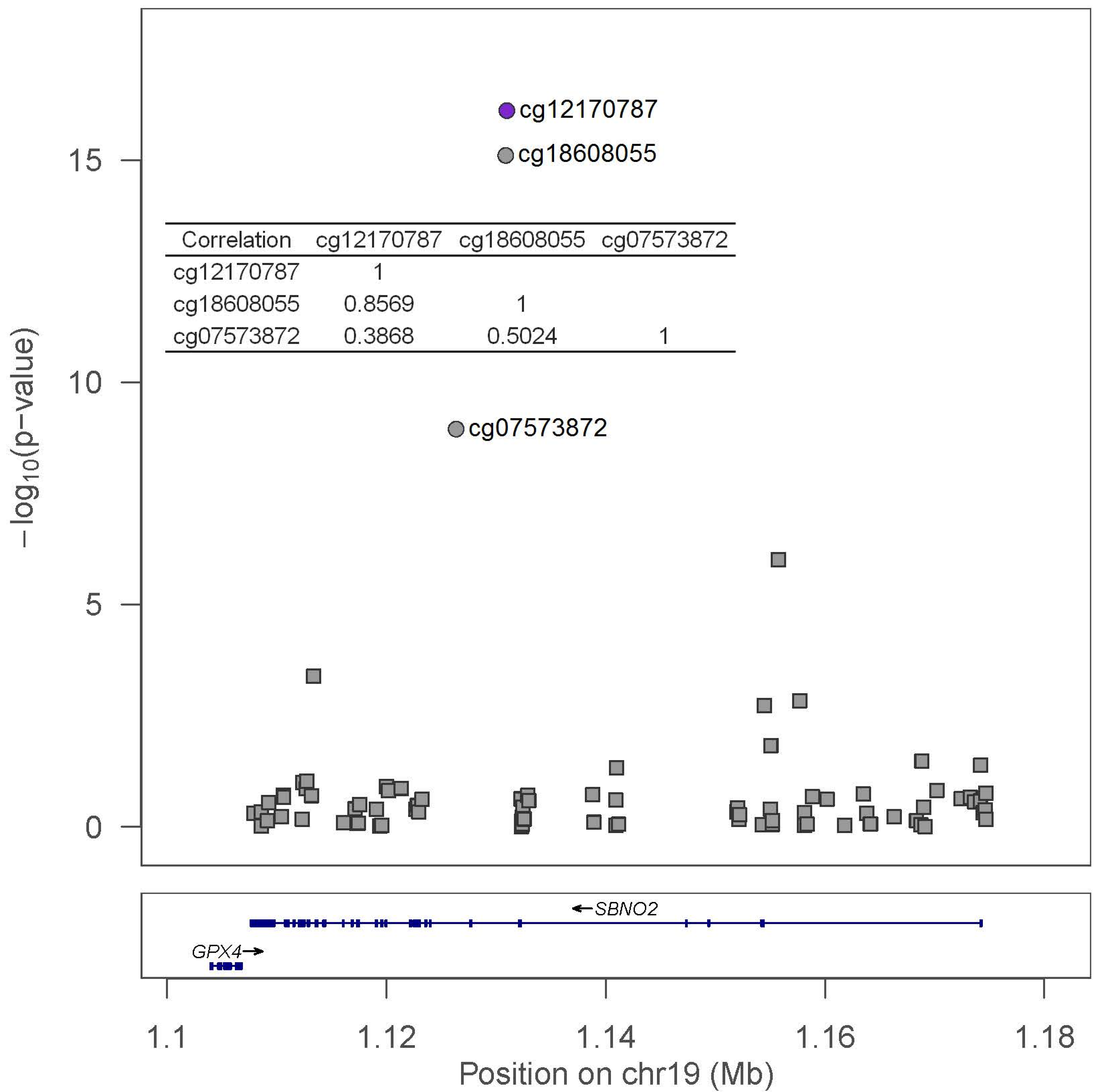

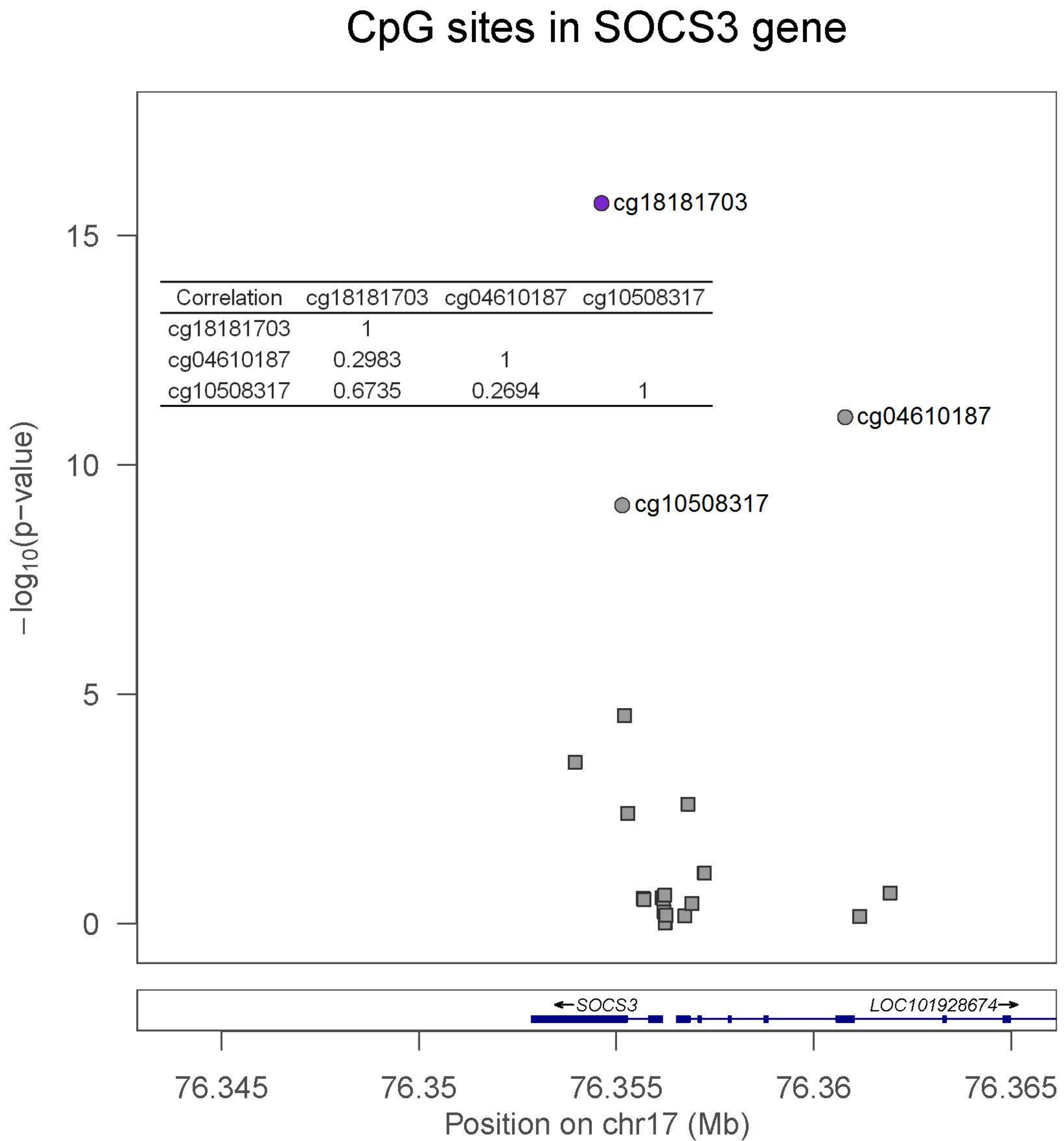

Correlation	cg18181703	cg04610187	cg10508317
cg18181703	1		
cg04610187	0.2983	1	
cg10508317	0.6735	0.2694	1

CpG sites in VMP1 gene

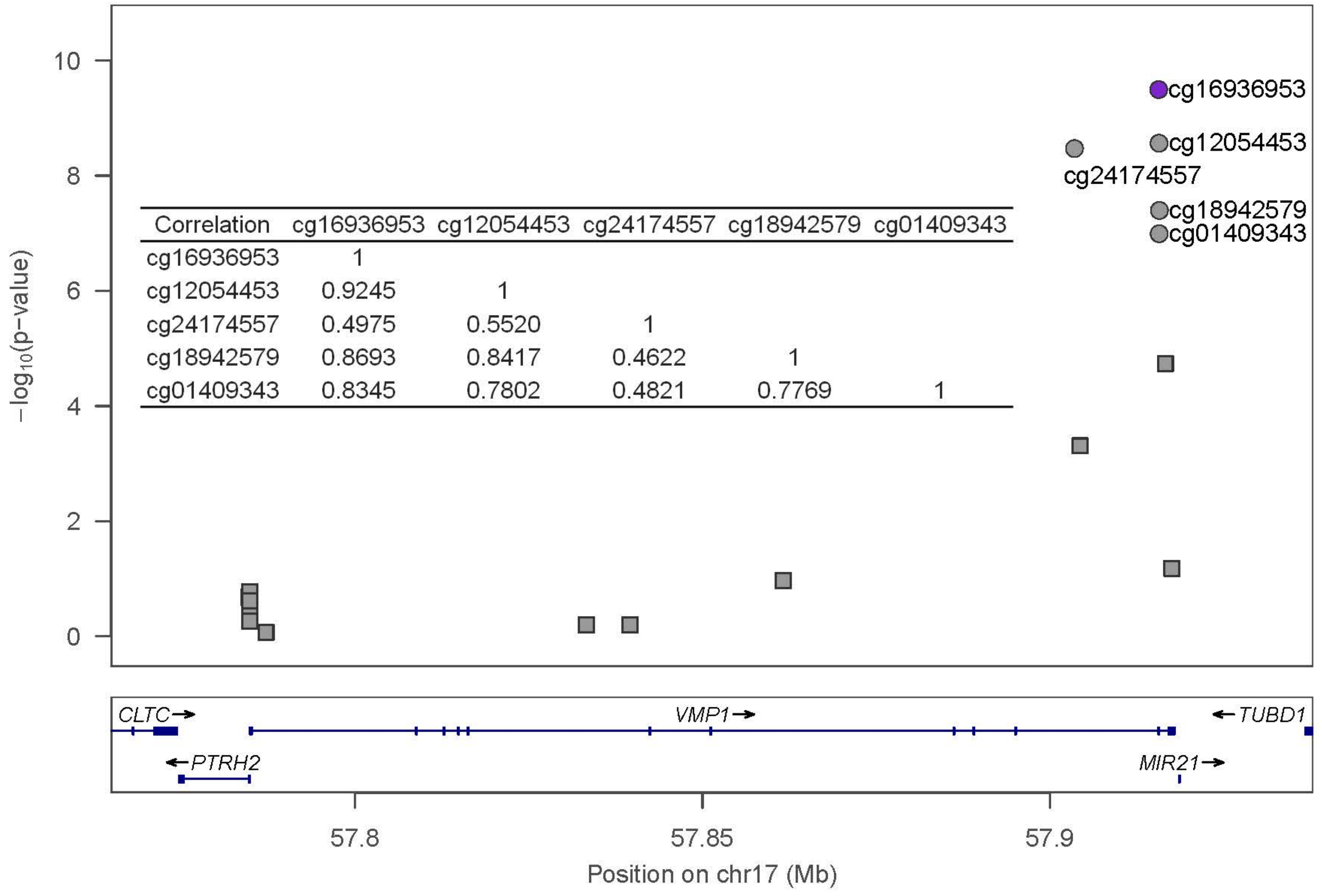

Correlation	cg16936953	cg12054453	cg24174557	cg18942579	cg01409343
cg16936953	1				
cg12054453	0.9245	1			
cg24174557	0.4975	0.5520	1		
cg18942579	0.8693	0.8417	0.4622	1	
cg01409343	0.8345	0.7802	0.4821	0.7769	1

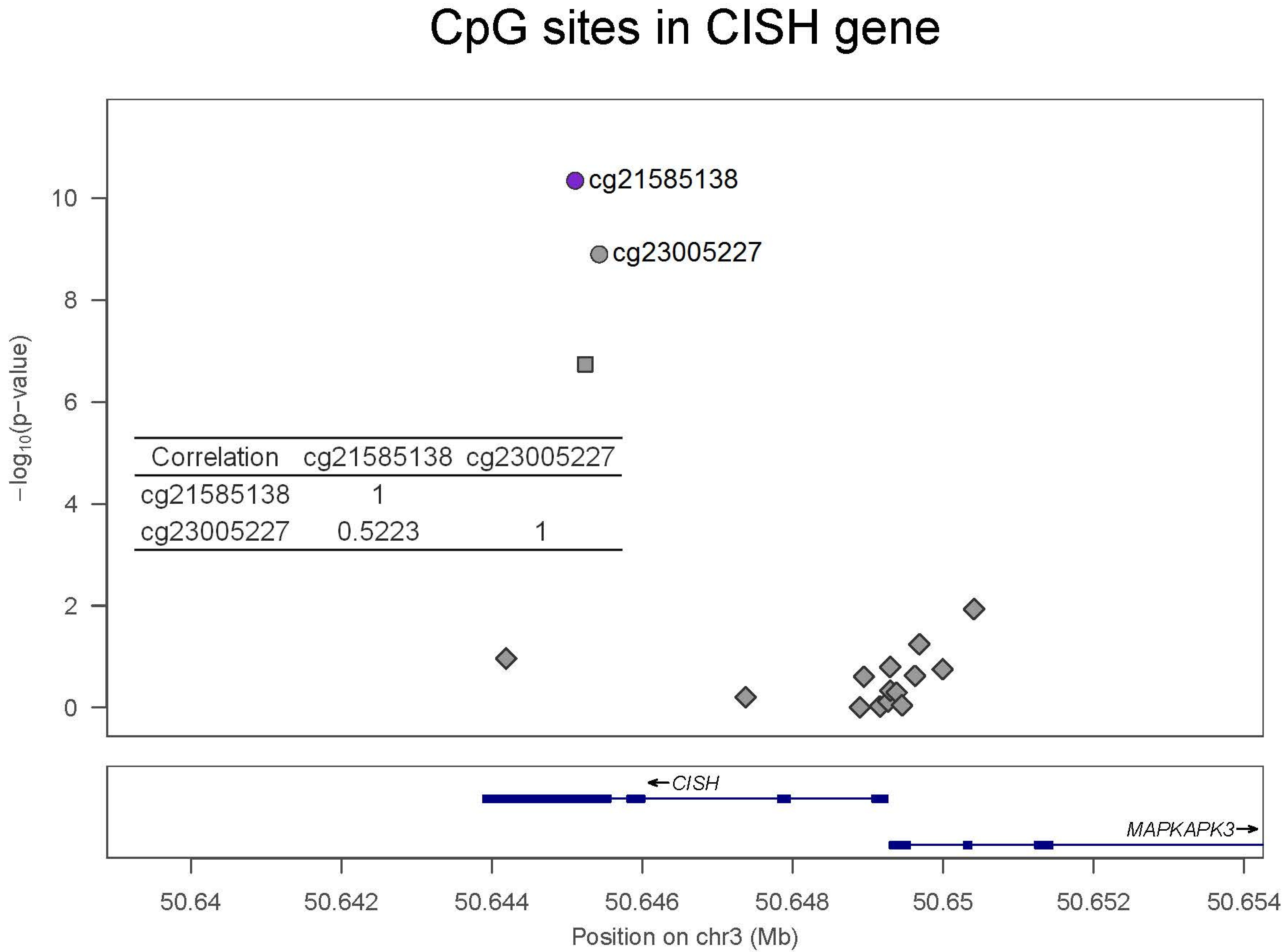
CpG sites in CISH gene
cg21585138
cg23005227
Correlation cg21585138 cg23005227
cg21585138 1
cg23005227 0.5223 1
−log10(p−value)
10
8
6
4
2
0
CISH
MAPKAPK3
50.64
50.642
50.644
50.646
50.648
50.65
50.652
50.654
Position on chr3 (Mb)
